# Sinusoidal Single-Pixel Imaging Based on Fourier Positive–Negative Intensity Correlation

**DOI:** 10.3390/s20061674

**Published:** 2020-03-17

**Authors:** Ling-Tong Meng, Ping Jia, Hong-Hai Shen, Ming-Jie Sun, Dong Yao, Han-Yu Wang, Chun-Hui Yan

**Affiliations:** 1 Changchun Institute of Optics, Fine Mechanics and Physics, Chinese Academy of Sciences, Changchun 130033, China; zy1517111@buaa.edu.cn (L.-T.M.); jiap@ciomp.ac.cn (P.J.); shenhh@ciomp.ac.cn (H.-H.S.); yaodong@ciomp.ac.cn (D.Y.); 2014204217@tju.edu.cn (H.-Y.W.); chh_yan@sina.com (C.-H.Y.); 2 Key Laboratory of Airborne Optical Imaging and Measurement, Chinese Academy of Sciences, Changchun 130033, China; 3 School of Instrumentation Science and Optoelectronic Engineering, Beihang University, Beijing 100191, China

**Keywords:** single-pixel imaging, computational ghost imaging, Fourier matrix, sinusoidal illumination patterns, positive–negative intensity correlation

## Abstract

Single-pixel imaging techniques extend the time dimension to reconstruct a target scene in the spatial domain based on single-pixel detectors. Structured light illumination modulates the target scene by utilizing multi-pattern projection, and the reflected or transmitted light is measured by a single-pixel detector as total intensity. To reduce the imaging time and capture high-quality images with a single-pixel imaging technique, orthogonal patterns have been used instead of random patterns in recent years. The most representative among them are Hadamard patterns and Fourier sinusoidal patterns. Here, we present an alternative Fourier single-pixel imaging technique that can reconstruct high-quality images with an intensity correlation algorithm using acquired Fourier positive–negative images. We use the Fourier matrix to generate sinusoidal and phase-shifting sinusoid-modulated structural illumination patterns, which correspond to Fourier negative imaging and positive imaging, respectively. The proposed technique can obtain two centrosymmetric images in the intermediate imaging course. A high-quality image is reconstructed by applying intensity correlation to the negative and positive images for phase compensation. We performed simulations and experiments, which obtained high-quality images, demonstrating the feasibility of the methods. The proposed technique has the potential to image under sub-sampling conditions.

## 1. Introduction

Optical cameras are some of the earliest and most commonly used remote sensing equipment. An optical camera generally comprises a lens unit and a photosensitive unit. In recent years, the photosensitive unit has changed from traditional films to electronic devices, such as the charge-coupled device (CCD) and the complementary metal oxide semiconductor (CMOS). The performance of a digital camera is determined by the performance of each pixel on the detector array. From mobile phones to professional, digital single-lens reflex (SLR) cameras, the number of pixels that make up a sensor chip is both a performance indicator and a marketing necessity. When multi-pixel detectors cannot be applied due to the weak signals caused by scattering or absorption losses, a single-pixel detector might be used as an alternative method to perform imaging. Researchers have shown that an image can be captured with just one pixel based on spatially structured illumination and multiple measurements in the time domain. This technique is called computational single-pixel imaging (SPI), also known as ghost imaging (GI) [1,2,3,4,5,6,7,8,9,10,11,12,13,14].

The first ghost imaging technique was proposed in 1995 [1], in which a photon-pair source was used to perform imaging. Later, it was experimentally demonstrated that ghost imaging can be accomplished using classical light sources [2,3], raising the question of whether ghost imaging is quantum exclusive. In 2008, a computational ghost imaging (CGI) scheme [4] was proposed based on classical coherent propagation theory, demonstrating that ghost imaging is not quantum necessary. The CGI uses a spatial light modulator (SLM) to generate programmable speckle patterns and projects structured illumination on the object. The image is reconstructed by recording only the total light intensities backscattered by the object surface in each projection of pre-known speckle patterns with a single-pixel detector. These intensities, respectively, correspond to the pre-defined modulating basis of the structured illumination patterns. The image is reconstructed by an iterative intensity correlation mathematical algorithm. In recent years, researchers became aware of the fact that the basis modulation can be performed as either structured illumination or focal plane modulation [15], the latter of which is more often referred to as single-pixel imaging [15,16,17,18,19,20,21,22,23,24,25,26,27,28,29,30,31,32]. SPI offers advantages in a growing range of non-conventional imaging applications such as wide spectrum imaging [16,17,18], depth mapping [19,20], and imaging with spatially variant and reconfigurable resolution [12,31,32].

SPI requires multiple measurements in the time domain to spatially reconstruct images, resulting in a long acquisition time. The minimum pixel number occupied by the aim target in an image is determined by the recognition algorithm and subjective judgment of human eyes. In the case of ensuring a certain spatial resolution, the selection of structured illumination patterns is the main factor affecting imaging time. Compared to random speckle patterns, the orthogonal basis is the best modulation basis of a light source for generating structured light in single-pixel imaging techniques in order to reduce the number of measurements. Hadamard single-pixel imaging and Fourier single-pixel imaging are two single-pixel imaging techniques with good performance in recent years [22,23,24,25,26,27,28,29]. Hadamard single-pixel imaging is mainly based on the Hadamard transform, while Fourier single-pixel imaging is mainly based on the Fourier spectrum acquisition and the inverse Fourier transform. In this paper, we propose a sinusoidal single-pixel imaging technique based on Fourier positive–negative intensity correlation. We used greyscale cosine patterns (the real part of the Fourier matrix) and sinusoidal patterns (the imaginary part of Fourier matrix). They were played on a projector and used for structured illumination instead of random speckle patterns. instead of random speckle patterns for illumination. In general, the proposed technique only needs sinusoidal illumination. Cosine illumination can be obtained by a one-step phase shift of sinusoidal illumination, so we refer to the technique as sinusoidal single-pixel imaging. Inspired by the discrete cosine transform (DCT) technique, which is mainly used to compress data or images with good decorrelation performance, it can transform signals from the spatial domain to the frequency domain. We convert the complex Fourier matrix’s modulation into intensity correlation, which is a process of image data compression and reproduction. The proposed technique has the potential to image under sub-sampling conditions with low-frequency sinusoidal illumination patterns.

## 2. Experiments and Methods

Although Hadamard single-pixel imaging and Fourier single-pixel imaging are the mainstream of SPI in recent years, we want to show a new application of orthogonal sinusoidal patterns in SPI. We use the Fourier matrix to generate sinusoidal and cosine structural illumination patterns, both of which can reconstruct images in the imaging course with symmetrical overlapping shadows due to missing phase information. A clear image is finally reconstructed by applying the intensity correlation to the negative and positive images for phase compensation [13,14,33,34],

In the past, positive and negative ghost imaging was a technology derived from the selection of the detector’s recording intensity data combined with a specific algorithm, which focused on data selection and algorithm programming. The positive and negative images obtained here are attributed to the inherent characteristic of the Fourier matrix. We named the phenomenon “Fourier positive–negative intensity correlation”. The proposed technique may bring new methods to SPI.

To generate orthogonal sinusoidal and cosine illumination patterns, we used the Fourier matrix. We verified the effectiveness of the proposed technique by numerical simulations and experimentation. We calculated the signal to noise ratio (SNR) of reconstructed images to evaluate the image quality. We used 16,384 two-dimensional (2D) basis images and 16,384 projective illumination patterns to obtain 128×128 spatial resolution images in simulations and experiments. Finally, we obtained clear images without an image-processing algorithm.

To generate orthogonal sinusoidal and cosine patterns, we needed to first generate the complex Fourier matrix. We defined a complex matrix F as the Fourier matrix, and it was no longer a member of the real number field $R^{n}$, but a member of the n-dimensional complex domain $C^{n}$, and every element in F was a complex number. The N×N square matrix F with entries is given by

(1) $$F_{jk}=e^{2\pi ijk/N}=w^{jk},$$

where j,k=0,1,2,⋯,N−1 and i is the imaginary number and is defined with the formula $i=\sqrt{-1}$, and normalized by $1/\sqrt{N}$ to make it a unitary matrix. The discrete N×N square Fourier matrix is defined below:

(2) $$F_{N}=\left[\begin{array}{cccc} w^{0} & w^{0} & \ldots & w^{0}\\ w^{0} & w^{1} & \ldots & w^{N-1}\\ \vdots & \vdots & \ddots & \vdots \\ w^{0} & w^{N-1} & \cdots & w^{\left(N-1)(N-1\right)} \end{array}\right]=\left[\begin{array}{cccc} 1 & 1 & \ldots & 1\\ 1 & w^{1} & \ldots & w^{N-1}\\ \vdots & \vdots & \ddots & \vdots \\ 1 & w^{N-1} & \cdots & w^{\left(N-1)(N-1\right)} \end{array}\right],$$

Euler’s formula is defined as follows:

(3) $$e^{ix}=\cos x+i\sin x,$$

where w is a member of the $N^{th}$ roots of unity ($\left\{1,w,w^{2},\cdots ,w^{n-1}\right\}$) and is defined with the formula

(4) $$w=e^{2\pi i/N}=\cos\frac{2\pi }{N}+i\sin\frac{2\pi }{N},$$

According to the properties of the unitary matrix, the number of measuring times required by applying the discrete Fourier matrix as the modulation matrix of the light source in SPI to reconstruct the complete image is equivalent to the spatial resolution (the number of pixels included in the image) of the image. In reality, we could not reproduce complex numbers in a single projection by structured light illumination patterns. As a result, we proposed a sinusoidal single-pixel imaging technique based on Fourier positive–negative intensity correlation. The imaging course is divided into two steps. First, two intensity images are obtained by modulating the structured light with sine and cosine functions. Secondly, a clear image is obtained by phase compensation of the two images. As mentioned above, the complex Fourier matrix is divided into two parts. The real part of the discrete N×N square Fourier matrix is made up of a cosine function and a row of the real number one. The imaginary part of the discrete N×N square Fourier matrix is made up of a sine function and a row of the real number zero. To transform the sine- and cosine-based modulation of structured light into a form that can be played on projectors, we extended the trigonometric functions’ values to the positive range. The family of trigonometric functions $\left\{1,\cos\frac{k\pi x}{l},\sin\frac{k\pi x}{l}\right\}$ satisfies the condition of orthogonal completeness. In the interval [−l,l], the following relationship is satisfied,

(5) $$\int_{-l}^{l}\cos\frac{k\pi x}{l}\sin\frac{k\pi x}{l}dx=0,$$

where k is a natural number.

To modulate the three-dimensional (3D) scene with sinusoidal illumination patterns and finally reconstruct 2D images, we needed to reshape the one-dimensional (1D) sine and cosine signals into a 2D format. We used the real part of a 128×128 square Fourier matrix to illustrate the operation methods.

**Figure 1:**
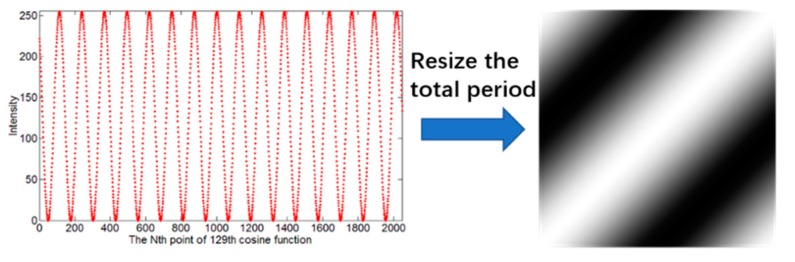
Resizing a row of the real part of the Fourier matrix to generate a 2D pattern.

As shown in Figure 1, to generate a grayscale 2D illumination pattern, a reshape method is applied to a 1D discrete cosine signal that is a row of the real part of the Fourier matrix. For example, there are 16,384 discrete points in a signal period. For ease of viewing, we intercept a one-eighth period from the 129th entire signal, and it is easy to concatenate these 2048 discrete points into a cosine signal through curve fitting. To reconstruct a 2D square image with the 1D cosine signal, we reshape the total period, generating a 128×128 pattern of pixels. We set the first 128 points on the first row, the 129th to 256th points on the second row and so on, until a square pattern is generated.

Defining f(x) as a period function in the interval [−l,l], we calculate the limit of the Fourier series of the defined function:

(6) $$f(x)=\underset{l\to \infty }{\lim}\{\frac{a_{0}}{2}+\sum_{k=1}^{\infty }(a_{k}\cos w_{k}x+b_{k}\sin w_{k}x\},$$

After estimating, it can be simplified as:

(7) $$f(x)=\int_{0}^{\infty }A(w)\cos(wx)dw+\int_{0}^{\infty }B(w)\sin(wx)dw,$$

where A(w) and B(w) are Fourier transform coefficients. They can be calculated by using integrals as below:

(8) $$\begin{array}{l} A(w)=\frac{1}{\pi }\int_{-\infty }^{\infty }f(\xi )\cos(w\xi )d\xi \\ B(w)=\frac{1}{\pi }\int_{-\infty }^{\infty }f(\xi )\sin(w\xi )d\xi \end{array}$$

The Fourier integrals in complex form can be derived from the above Fourier integrals in the real number field. We simulate the proposed sinusoidal SPI technique in mathematics based on the Fourier integrals above. To reconstruct an image of the target scene employing Fourier positive–negative intensity correlation, the sinusoidal structured light field modulated by the object surface can be expressed as:

(9) $$\begin{array}{l} F_{P}(x,y)=\langle I_{r-n}I_{r-n}(x,y)\rangle \\ F_{N}(x,y)=\langle I_{i-n}I_{i-n}(x,y)\rangle \\ F(x,y)=F_{P}(x,y)+F_{N}(x,y) \end{array}$$

where F(x,y) is the reconstructed image of the target, and $F_{P}(x,y)$ and $F_{N}(x,y)$ are the Fourier positive image and the Fourier negative image, respectively. $I_{r-n}(x,y)$ and $I_{i-n}(x,y)$ are the spatial intensity distributions of the Nth reshaped 2D Fourier real part pattern and the Nth reshaped 2D Fourier imaginary part pattern, respectively. 〈⋯〉 is the average algorithm for the items in parentheses. $I_{r-n}$ and $I_{i-n}$ are the intensities measured by the single-pixel detector after the Fourier positive–negative illumination patterns are modulated by the target surface, which can be expressed by the integrals:

(10) $$\begin{array}{l} I_{r-n}=\int_{light-field}O(x,y)I_{r-n}(x,y)dxdy\\ I_{i-n}=\int_{light-field}O(x,y)I_{i-n}(x,y)dxdy \end{array}$$

where O(x,y) is the target function. The proposed imaging technique is devoted to obtaining the exact target function by Equations (9)–(10). $I_{r-n}(x,y)$ and $I_{i-n}(x,y)$ can be substituted by the 2D sinusoidal pattern’s matrix according to the propagation theory of light field in free space. The other variable in Equation (10) is measured and recorded after each frame of illumination. Thus, F(x,y) can be reconstructed. We introduced root mean square error (RMSE) to compare the difference between the real target function O(x,y) and the reconstructed target function F(x,y) in simulations, and signal to noise ratio (SNR) in experiments. RMSE and SNR are calculated by the equations below [32]:

(11) $$\begin{array}{l} RMSE=\sqrt{\sum {\left[O(x,y)-F(x,y)\right]}^{2}/\left(M\times N\right)}\\ SNR=\left(\langle I_{S}\rangle -\langle I_{B}\rangle \right)/\left[\left(\sigma_{S}+\sigma_{B}\right)/2\right] \end{array}$$

where M and N are the numbers of spatial image pixels, $\langle I_{S}\rangle$ is the average intensity of the signal (in our experiment, we used the simple USAF 1951 resolution board as the target, and it was calculated from the bright white region we chose), $\langle I_{B}\rangle$ is the average intensity of the background (it was calculated from the dark black region we chose), and $\sigma_{S}$ and $\sigma_{B}$ are the standard deviations of the above regions, respectively.

## 3. Results

To evaluate the performance of the proposed technique, we prove it through simulations and experiments. We firstly generate a complex discrete 16,384×16,384 square Fourier matrix and separate it into two parts, the real cosine matrix and the imaginary sine matrix. Half of the cosine and sine matrix data are selected according to symmetry for subsequent calculation. We reshape the selected discrete sampling points into 2D square patterns and obtain 8192 grayscale cosine modulation patterns and 8192 grayscale sine modulation patterns. Their element values are normalized to the range of 0 to 255 for projecting, and each pattern corresponds to a coefficient for reconstruction. To keep dimension the same for calculation, we use 128×128-pixel images as the target in numerical simulations. We use a printed USAF 1951 resolution board as the target in experiments. We obtain Fourier positive images and Fourier negative images simultaneously in numerical simulations, which benefit from the software’s parallel computing. It is also convenient to obtain clear images with Fourier positive and negative images by phase compensation.

**Figure 2:**
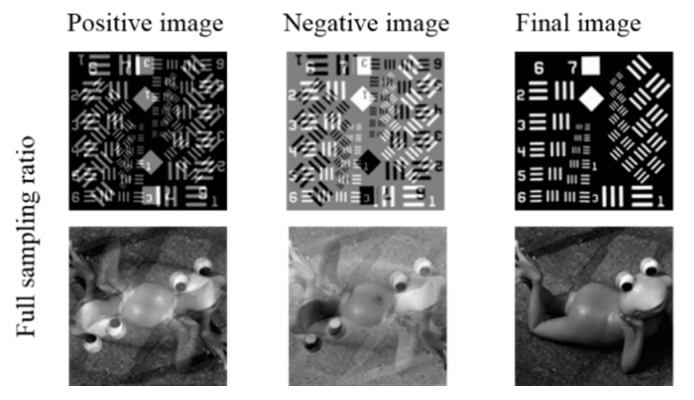
Positive and negative images with a full sampling ratio.

The imaging steps are shown in Figure 2, and we choose two targets as examples. The proposed technique obtains three images, and the final image is reconstructed by positive–negative images intensity correlation (we also call it phase compensation). The positive–negative images are the result of two overlapped images. The two overlapped images are made up of an image and its 180-degree rotated duplicate with different intensities. We have not found a method to directly extract the original image in a positive or negative image.

**Figure 3:**
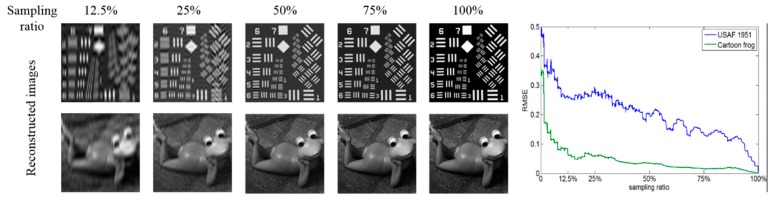
Reconstructed images and RMSE with different sampling ratios in the numerical simulation.

**Figure 4:**
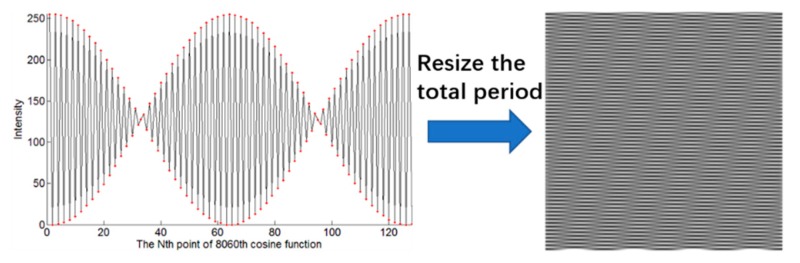
Insufficient sampling capacity of the Fourier matrix in high frequencies.

Any continuous signals can be expressed as infinite superpositions of sine wave signals with different frequencies. We apply this principle to a 2D space and propose the imaging technique. Different frequency information plays different roles in image structure. The main component of general images is low-frequency information, which forms the basic gray level of the image. High-frequency information forms the edges and details of the image. Therefore, it is possible to reconstruct images with low-frequency patterns and a low sampling ratio. The simulation results are shown in Figure 3. The resolution of the resulting image is 128×128 pixels, and the full sampling ratio means 16,384 measurements. We use RMSE to compare the quality of images resulting from the different sampling ratios. One of the curves is undulating obviously, and both of them show a downward trend, roughly. There are obvious ringing effects in the edges of the image when the sampling ratio is low. The direct cause of this effect is the loss of information in the process of imaging, especially the loss of high-frequency information. This seriously reduces the quality of the reconstructed image and makes it difficult to perform a subsequent image-processing algorithm on the image. We also noticed that the Fourier matrix has inherent flaws. In the high-frequency region, the phenomenon of insufficient sampling capacity is obvious. We choose the 8060th cosine signal, for example, and take the same approach as described above. We intercept 1/128 period from the 8060th entire signal, and we use MATLAB to connect the 128 discrete points (red points in Figure 4). It no longer seems like a cosine signal, although its generation is based on the cosine function. The reason for this is that in the high-frequency region, there are not enough sampling points to describe the cosine signal completely. To obtain clear result images when the sampling ratio is low, reordering the patterns by frequency may be effective. The reordering needs to pay attention to two points. The first is proposing a method to evaluate the high–low degree of the frequencies belonging to the patterns by complexity levels, and the second is choosing high-frequency patterns as accurately as possible.

**Figure 5:**
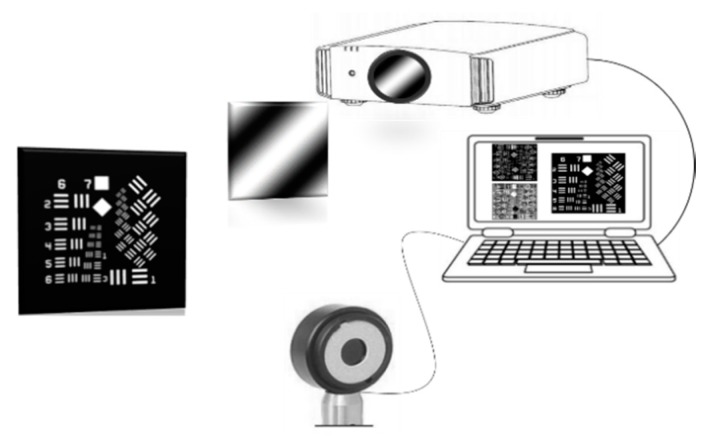
Schematic of the experimental set-up.

We used the natural order Fourier matrix to reconstruct a 128×128-pixel image in the experiment. As shown in Figure 5, there are no light sources except the commercial projector in the experimental set-up. The projector keeps the projection frequency at 6 frames per second. When the frame frequency is increased, frames are sometimes lost. We used a USAF 1951 resolution board as the target, printed on an A4 paper. To reconstruct a clear image, 16,384 illumination patterns are needed. We used 8192 cosine illumination patterns and the same number of sine illumination patterns to measure the reflected intensity by a photodiode, and the results were recorded on the laptop computer. We obtained positive and negative images, and a clear image was finally reconstructed by Fourier positive–negative intensity correlation. It took over 0.75 h to measure and record the intensities to obtain a perfect image under the above conditions. The change of lighting in the environment over such a long experiment time would have a certain influence on the experimental results. The black background of the printed target minimizes the interference of reflected stray light in the experiment. We also remained in the darkroom during the experiment to avoid interference from outside light sources.

**Figure 6:**
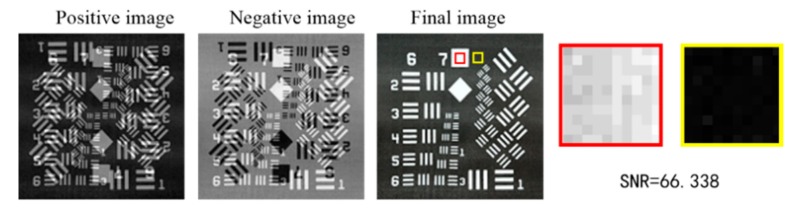
Experimental results based on Fourier positive–negative intensity correlation.

The images reconstructed by the proposed technique are shown as Figure 6. The SNR was calculated with Equation (11); the region of the signal is boxed with a red rectangle, and the region of the background is boxed with a yellow rectangle. There are some white stripes on the final image, with the main reason being the density inhomogeneity of the printed black background, which is especially obvious under the strong light of the projector. Excluding the imperfections of the experimental results, the imaging process and experimental phenomena are consistent with the numerical simulations.

## 4. Discussion

In this paper, we use the cosine and sinusoidal functions to modulate the illumination patterns in Fourier single-pixel imaging. The orthogonality of the illumination patterns is achieved by Fourier positive–negative intensity correlation, which we also call phase compensation. Using numerical simulations and experimental results, we provide the imaging process and phenomena, and the experiment results agree with the numerical simulations. We acquire three images based on the proposed technique, and a clear image is reconstructed by Fourier positive–negative intensity correlation of the other two images. The SNR of the results confirms the effectiveness of the proposed imaging technique.

Compared with the existing mature single-pixel imaging techniques, such as Hadamard single-pixel imaging (HSI) and Fourier single-pixel imaging (FSI) [23], the proposed technique can also reconstruct perfect images with full sampling ratios based on such grayscale illumination patterns as FSI and HSI. When the sampling ratio is less than 25%, the ringing effect of the reconstructed image is particularly obvious and the image quality is worse than the results shown in the present article [23]. The main reason for this lies in the pattern frequency used in the low sampling ratio. We choose the original sequence of the discrete Fourier matrix for measurement in the present article, rather than choosing low-frequency patterns.

To reduce the imaging time and capture high-quality images based on the proposed technique under low sampling ratios as shown in [23], the following aspects are worthy of further research. The ups and downs of the RMSE curve provide the possibility for the reordering of the illumination patterns based on the frequencies. The obvious ringing effect under the low sampling ratio requires one to obtain high-frequency information. The illumination mode based on the projector also needs to be replaced to reduce the imaging time. A binary device such as a digital micro-mirror device (DMD) is worthy of consideration, as it has been proved feasible to perform Fast Fourier single-pixel imaging via binary illumination [28,29]. In summary, we can introduce frequency-domain filtering in the proposed technique by pattern selection. The low frequencies and accurate high frequencies ensure good images, and we will concentrate on the reordering method and selection of illumination patterns in the next work.

## References

[1] Pittman T.B., Shih Y.H., Strekalov D.V. (1995). Optical imaging by means of two-photon quantum entanglement. Phys. Rev. A..

[2] Bennink R.S., Bentley S.J., Boyd R.W. (2002). “Two-Photon” Coincidence Imaging with a Classical Source. Phys. Rev. Lett..

[3] Gatti A., Brambilla E., Bache M. (2004). Ghost Imaging with Thermal Light: Comparing Entanglement and Classicalcorrelation. Phys. Rev. Lett..

[4] Shapiro J.H. (2008). Computational ghost imaging. Phys. Rev. A.

[5] Bromberg Y., Katz O., Silberberg Y. (2008). Ghost imaging with a single detector. Phys. Rev. A.

[6] Katz O., Bromberg Y., Silberberg Y. (2009). Compressive ghost imaging. Appl. Phys. Lett..

[7] Ferri F., Magatti D., Lugiato L.A. (2010). Differential Ghost Imaging. Phys. Rev. Lett..

[8] Sun B.Q., Welsh S.S., Edgar M.P., Shapiro J.H., Padgett M.J. (2012). Normalized ghost imaging. Opt. Express.

[9] Luana O., Juan S.T.G., Alessia P., Marco P. (2018). Time-resolved nonlinear ghost imaging. ACS Photonics.

[10] Luana O., Juan S.T.G., Luke P., Vittorio C., Antonio C., Jacob T., Robyn T., Alessia P., Marco P. (2020). Hyperspectral terahertz microscopy via nonlinear ghost imaging. Optica.

[11] Mahdi Khamoushi S.M., Nosrati Y., Hassan Tavassoli S. (2015). Sinusoidal ghost imaging. Opt. Lett..

[12] Sun M.J., Wang H.Y., Huang J.Y. (2019). Improving the performance of computational ghost imaging by using a quadrant detector and digital micro-scanning. Sci. Rep..

[13] Yu W.K., Yao X.R., Liu X.F., Lan R.M., Wu L.A., Zhai G.J., Zhao Q. (2016). Compressive microscopic imaging with “positive-negative” light modulation. Opt. Commun..

[14] Liu H.C., Yang H., Xiong J., Zhang S. (2019). Positive and Negative Ghost Imaging. Phys. Rev. Appl..

[15] Sun M.J., Xu Z.H., Wu L.A. (2018). Collective noise model for focal plane modulated single-pixel imaging. Opt. Lasers Eng..

[16] Welsh S.S., Edgar M.P., Bowman R. (2013). Fast full-color computational imaging with single-pixel detectors. Opt. Express.

[17] Radwell N., Mitchell K.J., Gibson G.M. (2014). Single-pixel infrared and visible microscope. Optica.

[18] Edgar M.P., Gibson G.M., Bowman R.W. (2015). Simultaneous real-time visible and infrared video with single-pixel detectors. Sci. Rep..

[19] Sun M.J., Edgar M.P., Gibson G.M. (2016). Single-pixel three-dimensional imaging with time-based depth resolution. Nat. Commun..

[20] Peng X., Zhao X.Y., Li L.J., Sun M.J. (2020). First-photon imaging via a hybrid penalty. Photonics Res..

[21] Stantchev R.I., Sun B.Q., Hornett S.M., Hobson P.A., Gibson G.M., Padgett M.J., Hendry E. (2016). Noninvasive, near-field terahertz imaging of hidden objects using a single-pixel detector. Sci. Adv..

[22] Sun M.J., Meng L.T., Edgar M.P. (2017). A Russian Dolls ordering of the Hadamard basis for compressive single-pixel imaging. Sci. Rep..

[23] Zhang Z.B., Wang X.Y., Zheng G.A. (2017). Hadamard single-pixel imaging versus Fourier single-pixel imaging. Opt. Express.

[24] Jiang H.Z., Zhu S.G., Zhao H.J. (2017). Adaptive regional single-pixel imaging based on the Fourier slice theorem. Opt. Express.

[25] Bian L.H., Suo J.L., Hu X. (2016). Efficient single pixel imaging in Fourier space. J. Opt..

[26] Sun M.J., Zhang J.M. (2019). Single-pixel imaging and its application in three-dimensional reconstruction: A Brief Review. Sensors.

[27] Zhang Z.B., Ma X., Zhong J.G. (2015). Single-pixel imaging by means of Fourier spectrum acquisition. Nat. Commun..

[28] Zhang Z.B., Wang X.Y., Zheng G.A. (2017). Fast Fourier single-pixel imaging via binary illumination. Sci. Rep..

[29] Sun M.J., Huang J.Y. (2018). Suppressing the noise in binarized Fourier single-pixel imaging utilizing defocus blur. Opt. Laser Eng..

[30] Chan W.L., Charan K., Takhar D. (2008). A single-pixel terahertz imaging system based on compressed sensing. Appl. Phys. Lett..

[31] Phillips D.B., Sun M.J., Taylor J.M., Edgar M.P., Barnett S.M., Gibson G.M., Padgett M.J. (2017). Adaptive foveated single-pixel imaging with dynamic super-sampling. Sci. Adv..

[32] Sun M.J., Edgar M.P., Phillips D.B., Gibson G.M., Padgett M.J. (2016). Improving the signal-to-noise ratio of single-pixel imaging using digital microscanning. Opt. Express.

[33] Huang P.S., Zhang S. (2002). Fast three-step phase-shifting algorithm. Appl. Opt..

[34] Lei S., Zhang S. (2009). Flexible 3-D shape measurement using projector defocusing. Opt. Lett..

